# Association between local public housing authority policies related to criminal justice system involvement and sexually transmitted infection rates

**DOI:** 10.1186/s40352-021-00156-4

**Published:** 2021-11-17

**Authors:** Jonathan Purtle, Erdal Tekin, Luwam T. Gebrekristos, Linda Niccolai, Kim M. Blankenship

**Affiliations:** 1 Department of Health Management & Policy, Drexel University Dornsife School of Public Health, 3215 Market St., Philadelphia, PA 19104 USA; 2 Department of Economics, American University, Washington, DC USA; 3 Department of Epidemiology, Yale School of Public Health, New Haven, CT USA; 4 Department of Sociology, American University, Washington, DC USA

**Keywords:** Housing, Criminal justice, HIV, Sexuality transmitted infections, Policy

## Abstract

The policies of U.S. local public housing authorities influence which populations have access to stable housing, an important resource for health. We assessed whether the restrictiveness of local public housing authority policies related to people with criminal justice histories—a population at high risk for HIV/STIs—were associated with HIV/STI rates at the local-level. An ecological analysis was conducted using data from 107 local public housing authority jurisdictions. The independent variable was a score that quantified the presence/absence of eight policies related to the ability of people with criminal justice histories to obtain and retain public housing. The dependent variables were county-level rates of HIV, gonorrhea, syphilis, and chlamydia. Ordinary least squares regression with state fixed effects was used. We find that the restrictiveness of housing authority policies towards people with criminal justice histories were significantly associated with higher HIV and gonorrhea rates, but not syphilis or chlamydia. For example, local housing authorities with a policy score more restrictive than the median score had an additional 6.05 cases of HIV per 100,000 population (32.9% increase relative to the mean rate) and 84.61 cases of newly diagnosed gonorrhea (41.3% increase). Local public housing authority policies related to people with criminal justice histories could affect HIV/STI risk at the population-level. These policies should be considered in studies and interventions at the intersection of housing, health, and justice involved populations.

**Supplementary Information:**

The online version contains supplementary material available at 10.1186/s40352-021-00156-4.

## Introduction

Housing is widely recognized as a social determinant of health (Howden-Chapman, 2004; Shaw, 2004), and of human immunodeficiency virus (HIV) and sexually transmitted infections (STIs) in particular (Aidala & Sumartojo, 2007). Housing insecurity can increase HIV/STI by, for example, disrupting social support networks, increasing exposure to sexually and socially dangerous living situations, and fragmenting access to HIV/STI testing and treatment (Aidala et al., 2005; German & Latkin, 2012; Niccolai et al., 2019; Weir et al., 2007). A recent ecological analysis, for example, found that U.S. county-level rates of eviction from renter-occupied households were positively associated with county-level rates of chlamydia and gonorrhea, independent of other county-level risk factors for these STIs (Niccolai et al., 2019).

While substantive research has documented associations between housing and health, little is known about the specific policies that contribute to these relationships. The policies of U.S. local public housing authorities might be particularly important. Public housing authorities provide subsidized housing with funds from the U.S Department of Housing and Urban Development (HUD), but have broad authority over the policies in their jurisdictions. Of particular importance are housing authorities’ *Admissions and Continued Occupancy Policies (ACOPs)*, which specify the circumstances under which applicants are eligible for, and can be evicted from, public housing (Curtis et al., 2013; Keene et al., 2018; Tran-Leung, 2015). ACOPS are updated and submitted to HUD at least every 5 years. Per federal statute (24 CFR §982.553), ACOPs contain provisions related to the housing eligibility of people with criminal justice histories and the consequences of tenants becoming involved with the criminal justice system. These policy provisions have potential implications for population health, and health equity in particular. Given that Black Americans are disproportionately more likely to have history of criminal justice involvement than people who are white in the United States (Bureau of Justice Statistics, 2016), and the potential benefits of subsidized public housing programs for health (Blakely et al., 2011; Denary et al., 2021; Fenelon et al., 2017; Shaw, 2004; Simon et al., 2017), policies that restrict access to public housing assistance on the basis of criminal justice system involvement could exacerbate health disparities between Black Americans and people who are White (Wildeman & Wang, 2017).

In a recent study, we quantified the restrictiveness of ACOP policy provisions related to criminal justice involvement in 152 public housing authority jurisdictions (blinded).We found that most ACOPs were more restrictive than required by U.S. federal law, that there was wide variation in policy restrictiveness across local jurisdictions, and there was no association between ACOP restrictiveness among local housing authorities in the same state. We also found no significant difference in the presence of policy provisions stating that arrest is sufficient evidence to prove engagement in criminal activity in ACOPs published before and after a federal policy change prohibiting the practice—suggesting the content of ACOPs changes minimally over time. Neither this study nor prior research about local housing authority ACOPs (Curtis et al., 2013; Keene et al., 2018; Tran-Leung, 2015), however, has examined associations between the policy provisions of ACOPs and health outcomes.

The current exploratory study begins to address this knowledge gap by assessing associations between the restrictiveness of U.S. local housing authority ACOP provisions related to people with criminal justice histories and county-level HIV/STI rates. The study examines a very specific and understudied domain of public policy related to the criminal justice system that could have implications for health and health equity. Methodologically, the study extends to the area of health and justice research the practice of transforming sets of qualitative policy indicators into continuous measures of policy exposure (Avery & Peffley, 2005; Banting & Kymlicka, 2013; Liebertz & Bunch, 2018; Phillips et al., 2012; Rincón-Gallardo Patiño et al., 2020; Vandevijvere et al., 2019).

## Methods

The independent variable was the restrictiveness of housing authorities’ ACOP policy provisions related to people with criminal justice histories. To characterize this policy exposure, we used the data from our aforementioned study (blinded). In short, the most recent publicly available versions of ACOPs were identified through internet searches and coded by multiple coders to establish inter-rater reliability. Exploratory factor analysis was conducted using 16 ACOP policy provisions related to criminal justice system involvement and then confirmatory factor analysis was conducted and a scale was created with eight ACOP policy provisions. Additional details about the factor analysis methods and the factor loadings of the eight policy provisions are in Supplemental file 1.

Given the exploratory nature of the study and absence of prior research about ACOP policy provisions and health outcomes, ACOP score was operationalized and analyzed three different ways. First, ACOP restrictiveness was treated as a continuous score ranging from 0 (least restrictive) to 8 (most restrictive). These scores were derived from our prior study (blinded). Second, ACOP restrictiveness was treated as a dichotomous variable in which each housing authority was classified according to whether or not its ACOP score was ≥ the median restrictiveness score of 5. Third, ACOP restrictiveness was treated as an ordinal variable in which each housing authority was classified according to the quartile rank of its ACOP score.

The dependent variables were annual rates of HIV prevalence and newly diagnosed cases of gonorrhea, syphilis, and chlamydia per 100,000 population for the counties of urban housing authorities’ jurisdictions. These data were obtained for the most recent year available at the time of the analysis from the U.S. Centers for Disease Control and Prevention’s AtlasPlus. We used HIV prevalence as opposed to incidence data for our main analysis because newly diagnosed HIV rates, which are similar to incidence, were low and missing for many counties. Thus, although incidence is generally a more appropriate measure of policy impact than prevalence, we used HIV prevalence for our main analysis to reduce random error. In secondary exploratory analysis we used newly diagnosed HIV rates as the dependent variable. HIV data were from 2017 and gonorrhea, syphilis, and chlamydia data were from 2016—the most recent data available in AtlasPlus at the time of the analyses. Supplemental file 2 lists the housing authorities included in the analysis, their corresponding measures of ACOP restrictiveness and county-level HIV/STI rates, and ACOP publication year.

Ordinary least squares regression models were used to assess associations between ACOP restrictiveness and HIV/STI rates. The models used state fixed effects to account for time-invariant differences between states that may be associated with both ACOP restrictiveness and HIV/STI rates. To enable the use of state fixed effects, analysis was limited to instances when there were ACOP scores for two or more housing authorities within the same state (*n* = 107). We were unable to use county fixed effects because there was only one housing authority per county. The models weighted ACOP restrictiveness measures by the population size of each housing authority’s jurisdiction. Using data from City Health Dashboard (Gourevitch et al., 2019) used in the previous study (blinded), we assessed correlations between ACOP restrictiveness score and racial/ethnic diversity as well as racial/ethnic neighborhood segregation in the limited sample of 107 housing authority jurisdictions. We explored these correlations because these variables were significantly correlated with ACOP restrictiveness score in the full sample of 157 ACOPs. We did not find that these variables were significantly correlated with ACOP restrictiveness score in the limited sample (*r* ≤ .16, *p* ≥ .13) and thus did not adjust for these jurisdiction characteristics in our regression models.

Separate models were run with ACOP restrictiveness operationalized each of the three ways. To assess whether the ACOP score was a stronger predictor of HIV/STI rates than individual policy provisions, each of the eight policy provisions used to create the score was treated as a dichotomous independent variable in a separate model and its association with county-level HIV/STI rates was assessed.

## Results

Among the 107 counties included in the analysis, the mean ACOP score was 4.00 (SD = 2.86) and the median was 5. ACOP publication year ranged from 2009 to 2018, the modal publication year was 2016 (20.7%), followed by 2017 (18.9%), the median publication year was 2017, and there was no information on publication year for 11.7% of the ACOPs. The mean rates of HIV/STIs per 100,000 were 18.39 for HIV, 204.78 for gonorrhea, 11.28 for syphilis, and 624.05 for chlamydia.

When the ACOP score was treated as a continuous variable, a one-point increase in ACOP score was significantly associated with an additional 1.14 cases of HIV (6.2% increase relative to the mean rate) and 11.20 cases of gonorrhea per 100,000 county population (5.5% increase relative to the mean rate) (Table 1). When ACOP score was treated as dichotomous variables operationalized as being above or below the median ACOP score, having an ACOP score above the median was significantly associated with an additional 6.05 cases of HIV (32.9% increase relative to the mean rate) and 84.61 cases of gonorrhea per 100,000 county population (41.3% increase relative to the mean rate). When ACOP score was operationalized as a four-level ordinal variable, having an ACOP score in the lowest quartile was significantly associated with 8.65 few cases of HIV per 100,000 county population (47.0% decrease relative to the mean rate) compared to an ACOP score in the highest quartile. There were no statistically significant associations between ACOP score and syphilis or chlamydia rates regardless of how ACOP restrictiveness was operationalized.

**Table 1:** Associations between the Restrictiveness of Local Housing Authority ACO Policies (ACOP) Towards People with Criminal Justice Histories and County Human Immunodeficiency Virus (HIV) and Sexually Transmitted Infection Rates per 100,000 County Population

	HIV Prevalence Rate Per 100,000			Gonorrhea Newly Diagnosed Rate Per 100,000			Syphilis Newly Diagnosed Rate Per 100,000			Chlamydia Newly Diagnosed Rate Per 100,000		
	n	B	SE	n	B	SE	n	B	SE	n	B	SE
ACOP Score	94	1.14***	0.40	104	11.20*	5.27	104	0.44	0.49	104	11.34	8.52
Median ACOP Score												
Below Median ACOP Score	44	−6.05**	2.45	47	84.61**	− 39.20	47	−3.62	3.47		−61.05	57.41
Above Median ACOP Score (Ref)	50	–	–	57	–	–	57	–	–	57	–	–
Quartile ACOP Score												
1st (Range = 0, 1)	28	−8.55***	3.01	30	−47.22	32.34	30	−2.34	2.71	30	−58.03	63.21
2nd (Range = 2, 5)	28	1.08	4.09	30	6.82	35.26	30	3.73	2.83	30	17.43	77.47
3rd (Range = 6)	15	1.16	3.67	17	86.35**	40.27	17	4.09	3.97	17	98.69	66.78
Ref: 4th (Range = 7, 8)	23	–	–	27	–	–	27	–	–	27	–	–
Specific ACOP Policy Provisions												
*Admission Decisions*												
Arrests and/or charges explicitly given less weight than conviction	52	−5.32**	2.35	55	8.91	25.6	55	1.91	2.25	55	25.91	55.74
Ref: Arrests and/or charges *not* explicitly given less weight than conviction	42	–	–	49	–	–	49	–	–	49	–	–
Mitigating circumstances explicitly considered	81	−2.02	2.86	88	−7.64	26.44	88	6.46^***^	2.19	88	−5.11	48.17
Ref: Mitigating circumstances *not* explicitly considered	13	–	–	16	–	–	16	–	–	16	–	–
Circumstances related to nature of the violation explicitly considered as mitigating circumstance	47	−3.90^*^	2.23	51	−57.65^*^	32.49	51	−1.42	2.69	51	− 29.02	55.18
Ref: Circumstances related to nature of the violation *not* explicitly considered as mitigating circumstance	47	–	–	53	–	–	53	–	–	53	–	–
Impact on family explicitly considered as mitigating circumstance	39	−8.30^***^	1.98	42	−98.66^***^	30.20	42	−4.54	2.89	42	− 119.97^**^	48.56
Ref: Impact on family *not* explicitly considered as mitigating circumstance	55	–	–	62	–	–	62	–	–	62	–	–
*Eviction Decisions*												
Family is explicitly permitted to remove member for any criminal/ drug use activity	46	−8.49^*^	2.30	52	−93.95^**^	39.94	52	−7.17^**^	3.23	52	−109.78^*^	56.00
Ref: Family is *not* explicitly permitted to remove member for any criminal/ drug use activity	48	–	–	52	–	–	52	–	–	52	–	–
Mitigating circumstances explicitly considered	56	−2.68	3.32	60	−46.45	33.78	60	−3.39	3.36	60	−61.61	55.37
Ref: Mitigating circumstances *not* explicitly considered	38	–	–	44	–	–	44	–	–	44	–	–
Proof of good tenancy explicitly considered as mitigating circumstance	29	−7.21^***^	2.52	31	−40.722	33.18	31	−4.30	2.84	31	−17.99	53.01
Ref: Proof of good tenancy *not* explicitly considered as mitigating circumstance	65	–	–	73	–	–	73	–	–	73	–	–
Impact on family explicitly considered as mitigating circumstance	34	−0.20	3.93	36	−38.98	27.33	36	−2.62	2.45	36	−56.75	49.98
Ref: Impact on family *not* explicitly considered as mitigating circumstance	60	–	–	68	–	–	68	–	–	68	–	–

^*^ *p* < .1, ^**^ *p* < .05, ^***^ *p* < .01. *HIV* Human immunodeficiency virus, *ACOP* Admissions and Continued Occupancy Policy. *Ref* Reference group. Standard errors are Huber/White corrected for arbitrary forms of heteroscedasticity

The restrictiveness score was generally a stronger predictor of HIV/STI rates than the individual policy provisions. However, for both HIV and gonorrhea, higher rates were strongly and significantly associated with the absence of an ACOP provision stating that the impact on an applicant’s family was considered as a mitigating circumstance in admissions decisions (B = − 8.30 and − 98.66, respectively) and the absence of an ACOP provision stating that a family is permitted to remove a member for any criminal/ drug use activity to avoid eviction (B = − 8.49 and − 93.95, respectively). In exploratory secondary analysis, there were no significant (*p* ≤ .05) associations between ACOP restrictiveness scores or provisions and newly diagnosed HIV rates. However, estimates were in the expected direction.

## Discussion

The restrictiveness of local housing authority policies towards people with criminal justice histories was consistently and significantly associated with higher HIV and gonorrhea rates. Although the ACOP restrictiveness score was generally more strongly associated with HIV and gonorrhea rates than individual ACOP provisions, some individual ACOP provisions (e.g., the impact of an admission denial because of a criminal justice involvement on the applicant’s family being considered as a mitigating circumstance in admission decisions) were strongly and significantly associated with HIV and gonorrhea. Future research is needed to understand how these policy provisions are implemented and affect the housing stability and behaviors of people with criminal justice histories and their families. This information could shed light on the specific pathways through which these policies could influence housing insecurity and HIV and gonorrhea risk. Additional research is also needed to explore why fairly consistent associations would be observed for HIV and gonorrhea but not syphilis or chlamydia.

The current study has limitations, many of which highlight areas for future research related to housing, health, and justice involved populations. Limitations of the study stem from the ecological nature of the county HIV/STI rates because the majority of the county population (i.e., the denominator used to calculate the rate) was likely to neither be eligible for public housing nor have a criminal justice history. Thus, only a small proportion of the county population was directly exposed to the ACOP policy provisions that were associated with HIV/STI rates. However, this misclassification bias would theoretically move the association towards the null and weaken, not strengthen, the magnitude of the observed associations. To address this limitation of ecological data in future research, studies conducted with people who have criminal justice histories could assess if and how housing authorities’ ACOP policy provisions are perceived as affecting HIV/STI risk behaviors (Blankenship et al., 2021).

The use of county-level HIV/STI data is also a limitation because the vast majority of housing authorities in the sample operate at the sub-jurisdictional city-level. County-level HIV/STI data were used because city-level HIV/STI data were not available for most housing authorities in the sample. Relatedly, HIV/STI rates by race and ethnicity were not widely available at the county-level. This prevented examination of associations between ACOP policy restrictiveness and the magnitude of HIV/STI disparities between Black Americans and people who are White within counties. Such associations would theoretically be stronger than those observed in the current study because Black Americans have greater exposure to ACOP policy provisions related to criminal justice involvement (because of structural racism which makes African Americans more likely to be involved with the criminal justice systems and meet the low-income requirements for public housing (Pogorzelski et al., 2005). The limitations of the data used in the current study highlight the need for HIV/STI data by race and ethnicity at sub-county (e.g., city) levels. These data are important for rigorously assessing the health impacts of local policies that exclude people with criminal justice histories from health promoting resources such as housing.

Finally, it should be emphasized that the study’s cross-sectional design restricts it from making any causal inferences about the effect of ACOP policy provisions on HIV/STI rates. This is especially true because ACOP publication year (exposure) did not precede the year of HIV/STI data (outcome) for some housing authority jurisdictions. As previously noted, however, there is little evidence that ACOP provisions related to people with criminal justice histories change over time.

## Conclusions

Local public housing authority policies related to the admission and eviction of people with criminal justice histories could potentially affect HIV/STI risk at the population-level. The use of summary scores that capture the presence/absence of multiple policy provisions may be a more precise indicator of policy exposure than the use of individual policy provisions in isolation. ACOP policies should be considered in studies and interventions related to housing, health, and justice involved populations.

## Supplementary Information

**Additional file 1: Supplemental file 1.** Factor Analyses Method Details.

**Additional file 2.**

## Data Availability

The datasets used and/or analyzed during the current study are available from the corresponding author on reasonable request.
