# Supplementary material for: Association between local public housing authority policies related to criminal justice system involvement and sexually transmitted infection rates
Source: Health Justice. 2021 Nov 17;9:32. doi: 10.1186/s40352-021-00156-4 (PMC8597229; doi:10.1186/s40352-021-00156-4)
Supplement: Supplementary file 1 — Additional file 1: Supplemental file 1. Factor Analyses Method Details. [file 40352_2021_156_MOESM1_ESM.doc]

**Supplemental File 1:**

**Factor Analyses Method Details**

We first investigated the underlying factor structure of 16 policy provision variables (Table 1 in main text) through exploratory factor analysis (EFA). We then verified the factor structure through confirmatory factor analysis (CFA). EFA and CFA was applied using the R packages psych and lavaan, respectively (Revelle, 2017; Rosseel et al., 2015). The overall Kaiser-Meyer-Olkin statistic was 0.75 (range= 0.39, 0.88), indicating that the data were suitable for factor analysis. All items were correlated with two or more items (*r* ≥ 0.3) To avoid confirmatory bias, the sample of coded ACOPs (n=152) were randomly divided into two samples, an EFA sample (n=76) and a CFA sample (n=76).

With the EFA sample, we computed a correlation matrix using polychoric and polyserial correlations between paired categorical and paired continuous and categorical variables, respectively. Because estimating the polychoric correlations tends to result in a non-positive definite correlation matrix and a positive definite matrix is necessary for factor analysis, we first used Bentler and Yuan’s smoothing algorithm to transform the correlation matrix into a positive definite matrix using R package psych (Bentler & Yuan, 2011; Revelle, 2017). To determine the number of factors to retain, we examined three indices: the ratio of the two highest eigenvalues, the scree plot, and parallel analysis (Carmines & Zeller, 1979; Cattell, 1966; Horn, 1965). Further, we eliminated factors with fewer than three factor loadings. Additionally, we removed variables that had a low factor loading (<0.45) or cross-loading. Finally, a CFA was used to determine the goodness of fit of the factor structure with the CFA sample.

EFA resulted in a single factor structure. A total of 8 variables were eliminated due to low factor loadings. Initially, the parallel analysis suggested a 5 factor structure. Upon further review, the third, fourth and fifth factors had fewer than 3 factor loadings and were therefore eliminated from the model. With the removal of the third, fourth and fifth factors, 5 variables had low factor loadings and were eliminated from the model. For the final EFA model, an additional 3 variables were eliminated, and a single factor solution was selected because: 1) 71% of the total variance was explained with 1 factor, 2) the eigenvalue ratio was greater than 4.0, which indicates unidimensionality, and 3) the eigenvalues on the scree plot plateaued after the first factor, suggesting a single factor solution (Carmines & Zeller, 1979; Cattell, 1966).

CFA was conducted with the second sample (n=76) under the hypothesis that a single factor is represented by 8 variables. Multiple model fit statistics indicated good fit: Root Mean Square Error of Approximation=0.04 (*p*=0.53) and Comparative Fit Index= 0.998, and χ^2^ *p*= 0.31. Factor loadings ranged from 0.65 to 0.99 (Table).

**Table: Eight Policy Provisions That Characterize the Restrictiveness of Local Housing Authority Policies Towards People with Criminal Justice Histories, Codified in Admission and Continued Occupancy Policies (ACOPs), N= 76**

| **ACOP Policy Provision** | **Factor Loading^1^** |
| --- | --- |
| Arrests and/or charges explicitly given less weight than conviction (admissions) | 0.68 |
| Mitigating circumstances explicitly considered (admissions) | 0.65 |
| Family is explicitly permitted to remove member for any criminal/ drug use activity (eviction) | 0.78 |
| Mitigating circumstances explicitly considered (eviction) | 0.87 |
| Circumstances related to nature of the violation explicitly considered as mitigating circumstance (admissions) | 0.93 |
| Impact on family explicitly considered as mitigating circumstance (admission) | 0.92 |
| Proof of good tenancy explicitly considered as mitigating circumstance (eviction) | 0.97 |
| Impact on family explicitly considered as mitigating circumstance (eviction) | 0.99 |

1. Results confirmatory factor analysis. Factor loadings based on a single factor solution.

**Factor Analysis Methods References**

Bentler, P. M., & Yuan, K.-H. (2011). Positive Definiteness via Off-Diagonal Scaling of a Symmetric Indefinite Matrix. *Psychometrika*, *76*(1), 119–123. https://doi.org/10.1007/s11336-010-9191-3

Carmines, E. G., & Zeller, R. A. (1979). *Reliability and validity assessment* (Vol. 17). Sage publications.

Cattell, R. B. (1966). The scree test for the number of factors. *Multivariate Behavioral Research*, *1*(2), 245–276.

Horn, J. L. (1965). A rationale and test for the number of factors in factor analysis. *Psychometrika*, *30*(2), 179–185.

Revelle, W. R. (2017). *psych: Procedures for personality and psychological research*.

Rosseel, Y., Oberski, D., Byrnes, J., Vanbrabant, L., Savalei, V., Merkle, E., … Barendse, M. (2015). Package ‘Lavaan’: Latent Variable Analysis. *R Package. Available Online at: Http://Lavaan. Org*.
